# Supplementary material for: MetaRibo-Seq measures translation in microbiomes
Source: Nat Commun. 2020 Jun 29;11:3268. doi: 10.1038/s41467-020-17081-z (PMC7324362; doi:10.1038/s41467-020-17081-z)
Supplement: Supplementary file 10 — Supplementary Data 7 [file 41467_2020_17081_MOESM10_ESM.zip › File2/Confidence_VeryHigh_Taxonomy/294010_out.krona.html]

Javascript must be enabled to view this page.

members
magnitude
magnitudeUnassigned
count
unassigned
taxon
rank

294010\_out

16

superkingdom
2
16

16
976
phylum

16
200643
class

16
171549
order

family
1853231
1

1
283168
genus

1897010
species

SRS148159\_contig\_number\_52599
1

12
family
171552

11
577309
genus

species
454154
9

SRS013687\_contig\_number\_17747SRS015782\_contig\_number\_contig-100\_150.244843SRS017307\_contig\_number\_31933SRS054352\_contig\_number\_20493SRS063985\_contig\_number\_21599SRS1041130\_contig\_number\_2342SRS105082\_contig\_number\_21010SRS1055022\_contig\_number\_16324SRS143876\_contig\_number\_14866

1263095
species

SRS023914\_contig\_number\_2306
1

1

SRS1041134\_contig\_number\_6640
species
454155

1
genus
838

species
165179
1

SRS012849\_contig\_number\_24663

3
815
family

816
genus
3

species
1907658
3

SRS015663\_contig\_number\_23808SRS051031\_contig\_number\_30962SRS893285\_contig\_number\_2021
